# What is the role of transformational leadership, work environment and patient safety culture for person‐centred care? A cross‐sectional study in Norwegian nursing homes and home care services

**DOI:** 10.1002/nop2.592

**Published:** 2020-08-13

**Authors:** Eline Ree

**Affiliations:** ^1^ SHARE – Centre for Resilience in Healthcare Faculty of Health Sciences University of Stavanger Stavanger Norway

**Keywords:** home care services, job demands, job resources, nursing homes, patient safety culture, person‐centred care, transformational leadership

## Abstract

**Aim:**

To examine how transformational leadership, job demands, job resources and patient safety culture contribute in explaining person‐centred care in nursing homes and home care services.

**Design:**

Cross‐sectional study.

**Methods:**

Healthcare professionals in four Norwegian nursing homes (*N* = 165) and four home care services (*N* = 139) participated in 2018. Multiple regression analyses were used to examine to what degree transformational leadership, job demands, job resources and patient safety culture dimensions predicted person‐centred care.

**Results:**

Transformational leadership, job demands and job resources explained 41% of the variance in person‐centred care, with work pace as the strongest predictor (*β* = 0.39 *p* < .001). The patient safety culture dimensions explained 57.5% of the variance in person‐centred care, with staffing being the strongest predictor (*β* = 0.31 *p* < .001). There were small differences between nursing homes and home care. In total, transformational leadership, pace of work, staffing and factors related to communication were the strongest predictors for person‐centred care.

## 1 INTRODUCTION

In healthcare research, policies and regulations, there is an increasing focus on person‐centred care, emphasizing its importance for healthcare quality (Coulter, 2006; Doyle, Lennox, & Bell, 2013; Ministry of Health & Care Services, 2018; World Health Organization, 2016). The institute of medicine (2001) identifies person‐centred care as one of six domains of healthcare quality and defines it as “care that is respectful of and responsive to individual patient preferences, needs, and values and ensuring that patient values guide all clinical decisions.” Systematic literature reviews have found that patient‐centred care was associated with clinical effectiveness, and better patient care processes and outcomes such as patient satisfaction, self‐management and improved quality of life (Doyle et al., 2013; Kim & Park, 2017; Rathert, Wyrwich, & Boren, 2013). Furthermore, a systematic review and meta‐analysis by Goldfarb, Bibas, Bartlett, Jones, and Khan (2017) revealed that patient‐centred care interventions in intensive care units resulted in decreased length of stay and improvement in several health‐related outcomes, including patient and family satisfaction, achievement of medical goals and mental health outcomes. Managers are facilitators for the implementation of person‐centred care interventions in healthcare settings (Beckett et al., 2013; Moore et al., 2017; Wijk, Åberg Jönsson, & Lindberg, 2019), by acting as role models, encouraging person‐centred care and initiating organizational change (Moore et al., 2017). A systematic review concluded that barriers to person‐centred care were related to lack of understanding (e.g. “competing roles of control over patient care”), organizational barriers (e.g. “daily rushed workloads”), individual barriers (i.e. “time constraints”) and interdisciplinary barriers (e.g. “communication barriers between doctors and nurses) (Kiwanuka, Shayan, & Tolulope, 2019).

Organizational structures and contextual factors play an important role in patients’ experience of care and can act as barriers for improvement at all levels of the system (Ocloo et al., 2020). A systematic review concludes that sound organizational and workplace cultures are associated with several patient outcomes, such as increased patient satisfaction, less falls and reduced mortality (Braithwaite, Herkes, Ludlow, Testa, & Lamprell, 2017). Similarly, the patient safety culture is related to a range of outcomes in different healthcare settings, including better patient experiences (Abrahamson, Hass, Morgan, Fulton, & Ramanujam, 2016; Najjar, Nafouri, Vanhaecht, & Euwema, 2015; Wang et al., 2014). Previous research argues for the importance of managers in creating a sound patient safety culture and quality of care (Merrill, 2015; Ree & Wiig, 2019b; Sfantou et al., 2017). The transformational leadership style is related to a range of positive organizational outcomes and processes in health care, including work environmental factors and patient safety culture (Boamah, Laschinger, Wong, & Clarke, 2018; Merrill, 2015; Ree & Wiig, 2019b). Transformational leadership can be defined as leaders that “broaden and elevate the interests of their employees, generate awareness and acceptance of the purposes and mission of the group, and stir their employees to look beyond their own self‐interest for the good of the group” (Bass, 1991, p. 21).

### 1.1 Background

Based on the existing literature, there are reasons to believe that transformational leadership, work environmental factors and patient safety culture influence the level of person‐centred care in healthcare settings. However, no previous studies have explored these associations. Furthermore, most research is conducted in the specialist healthcare setting, and there is a need for more knowledge about person‐centred care in nursing homes and home care services. Therefore, the current study will contribute with new and original knowledge about the role of leadership, work environment and patient safety culture for person‐centred care in these important healthcare settings, suggesting implications relevant to nursing management.

The overall aim of this study was therefore to explore how transformational leadership, job demands, job resources and patient safety culture contribute in explaining person‐centred care in nursing homes and home care services. The following research question was addressed: *To what degree can transformational leadership, job demands, job resources and patient safety culture explain person‐centred care in nursing homes and home care services?* We hypothesized that

1. Transformational leadership and job resources have a positive impact on person‐centred care, while job demands have a negative impact
2. Transformational leadership is a stronger predictor for person‐centred care than job demands and job resources
3. The patient safety culture dimensions have a positive impact on person‐centred care

## 2 METHODS

This study used a cross‐sectional design and is a part of the intervention project “Improving Quality and Safety in Primary Care—Implementing a Leadership Intervention in Nursing Homes and home care” (SAFE‐LEAD Primary Care) (Johannessen et al., 2019; Wiig et al., 2018).

### 2.1 Recruitment

A survey was conducted electronically among healthcare personnel from four nursing homes (*N* = 165) and four home care services (*N* = 139) in south‐western Norway in October 2018 using SurveyXact (Table 1). All employees who were employed in one of the units and fulfilled the inclusion criteria of being employment in at least 30% position and be able to read and write Norwegian were approached. The response rates were 65% (nursing homes) and 67.5% (home care). The units were located in five different municipalities and were strategically selected based on criteria such as variations in size and location (urban and rural). Nurse counsellors from the development centres for nursing homes and home care services in three Norwegian counties employed as co‐researchers in the SAFE‐LEAD project, assisted in the recruitment of units.

**TABLE 1:** Municipality characteristics and response rates of participating units in home care services (HC) and nursing homes (NH)

Units	Municipality characteristics (ca *N* of inhabitants)	Surveys completed *N* (response rate)
HC 1	Medium‐sized district (15–20,000)	65 (86.6%)
HC 2	Medium‐sized city (70–75,000)	22 (56.0%)
HC 3	Small district (<5,000)	26 (56.0%)
HC 4	Rural district (5–10,000)	25 (53.2%)
HC total		139 (67.5%)
NH 1	Large city, centre of the municipality (130–135,000)	90 (73.2%)
NH 2	Medium‐sized city (70–75,000)	23 (69.7%)
NH 3	Small district (< 5,000)	26 (45.0%)
NH 4	Rural district (5,000–10,000)	26 (65.0%)
NH total		165 (67.5%)
Total		304 (66.2%)

### 2.2 Sample

The total sample consisted of 94.1% females, with 92.1% females in nursing homes and 96.4% in home care. The distribution of age, occupational status and years of employment was relatively similar between the nursing home and home care samples (Table 2). The majority of the samples had high school education or higher education. Home care had a higher percentage of assistants (8.6%) compared with nursing homes (1.8%). A larger proportion of the sample in nursing homes had been employed 21 years or more (20%) compared with home care (8.6%).

**TABLE 2:** Descriptive sample information in nursing homes (*N* = 165) and home care (*N* = 139)

Background variables	Home care *N* (%)	Nursing homes *N* (%)
Age		
20–29 years	14 (10.1)	20 (12.1)
30–39 years	33 (23.7)	39 (23.6)
40–49 years	40 (28.8)	26 (15.8)
50–59 years	38 (27.3)	50 (30.3)
60+ years	14 (10.1)	30 (18.2)
Occupational status		
Managerial position	9 (6.5)	11 (6.7)
Healthcare workers with a minimum of bachelor degree	59 (42.4)	67 (40.6)
Healthcare workers, upper secondary school	56 (40.3)	79 (47.9)
Assistants	12 (8.6)	3 (1.8)
Other	3 (2.1)	5 (3.0)
Years of employment in current workplace		
<1 year	6 (4.3)	20 (12.1)
1–5 years	38 (27.3)	41 (24.8)
6–10 years	35 (25.2)	27 (16.4)
11–15 years	19 (13.7)	24 (14.5)
16–20 years	29 (20.9)	20 (12.1)
>21 years	12 (8.6)	33 (20.0)

Note

The table is previously reported in Ree and Wiig (2019a).

### 2.3 Questionnaire

The questionnaire consisted of questions and instruments related to patient safety culture, work environment, leadership and person‐centred care, and background information such as age, occupational status and years of employment. The instruments described below were used to explore the research questions in this study.

*Person‐centred care* was measured with the Person‐centered Care Assessment Tool (P‐CAT) (Edvardsson, Fetherstonhaugh, Nay, & Gibson, 2010). The instrument has proven to have sound psychometric properties and test–retest stability when tested in Norwegian residential units for older people (Rokstad, Engedal, Edvardsson, & Selbæk, 2012). The instrument consists of 13 statements about person‐centred care, measured on 5‐point Likert scales (1= “disagree completely” to 5= “agree completely”). Examples of statements were “Users are offered the opportunity to be involved in individualized everyday activities” and “We are free to alter work routines based on users’ preferences.” One of the items (“Residents are able to access outside space as they wish”) was removed in our study as we regarded that it did not fit with the study settings, especially not home care. The wording of some statements in the home care services survey was slightly modified, such as replacing “residents” with “users,” to fit the home care setting. The Cronbach's alpha in the current study was 0.836.

*The Nursing Home Survey on Patient Safety Culture* (NHSOPSC) was used to measure patient safety culture (Cappelen, Aase, Storm, Hetland, & Harris, 2016; Sorra, Franklin, & Streagle, 2008). We used a Norwegian version, validated in Norwegian nursing homes by Cappelen et al. (2016). In the home care version, the wording on some of the items was slightly modified to fit the home care context (e.g. “users” instead of “patients” and “unit” instead of “nursing home”). The Norwegian validation study found acceptable fit for the following 10‐factor solution of the scale (Cappelen et al., 2016) (Cronbach's alpha is given for the current sample):

- “Teamwork” (α = 0.821), for example “Staff feel like they are part of a team”
- “Staffing” (α = 0.821), for example “We have enough staff to handle the workload”
- “Compliance with procedures” (α = 0.53), for example “Staff follow standard procedures to care for patients”
- “Training and skills” (α = 0.656), for example “Staff get the training they need in this nursing home/unit”
- “Non‐punitive responses to mistakes” (α = 0.655), for example “Staff are blamed when a patient is harmed”
- “Handoffs” (α = 0.782), for example “We have all the information we need when patients are transferred from the hospital (medical information)”
- “Feedback and communication about incidents” (α = 0.781), for example “When staff report something that could harm a patient someone takes care of it”
- “Communication openness” (α = 0.811), for example “Staff ideas and suggestions are valued in this nursing home/unit”
- “Supervisor expectations and actions promoting patient safety” (α = 0.879), for example “My supervisor listen to staff ideas and suggestions about patient safety”
- “Management and organizational learning” (α = 0.802), for example “This nursing home/home care is always doing things to improve patient safety”

All items were rated on five‐point Likert scales from 1 (“never” or “totally disagree”) to 5 (“always” or “totally agree”).

*Transformational leadership* was measured with the Global Transformational Leadership Scale (GTL) (Carless, Wearing, & Mann, 2000). A study on a large sample of Norwegian employees found that the scale had sound psychometric properties (Nielsen, Skogstad, Gjerstad, & Einarsen, 2019). The instrument consists of seven items describing the following transformational leadership behaviours: communicates a vision, develops staff, provides support, empowers staff, is innovative, leads by example and is charismatic (Carless et al., 2000). All items were rated on 5‐point Likert scales from 1 = never to 5 = very often. Examples of items were “Encourages thinking about problems in new ways and questions assumptions” and “Treats staff as individuals, supports and encourages their development”. The scale has proven to have satisfactory reliability and convergent and discriminant validity (Carless et al., 2000). The Cronbach's alpha in the current study was 0.944.

*Job demands and job resources* were measured with the Short Inventory to Monitor Psychosocial Hazards (SIMPH) (Notelaers, De Witte, Van Veldhoven, & Vermunt, 2007). The instrument is based on the Job Demands–Resources model (Demerouti, Bakker, Nachreiner, & Schaufeli, 2001) and consists of 24 items rated on 4‐point Likert scales from 1 (“never”) to 4 (“always”). The instrument can be sorted in three job demands dimensions and three job resources dimensions. The dimensions are described in the following together with Cronbach's alpha values for the current sample. Job demands:

“Pace of work” (four items, α = 0.913), for example “Do you work under time constraints?”
“Mental workload” (three items, α = 0.778), for example “Do you have to focus your attention on several things simultaneously?”
“Emotional workload” (five items, α = 0.760), for example “Is your work heavy from an emotional viewpoint?”
Job resources:
“Skill utilization” (four items, α = 0.767), for example “Do you feel that you achieve something meaningful in your job?”
“Autonomy” (four items, α = 0.710), for example “Do you have an influence on the pace of work?”
“Participation” (four items, α = 0.850), for example “Can you participate in decisions affecting areas related to your work?”

### 2.4 Data analyses

We had no missing data since respondents had to answer each question before moving on to the next in the electronically survey. All analyses were conducted using IBM SPSS Statistics version 25.

Hierarchical multiple regression analyses were used to examine the impact of transformational leadership, job demands and job resources on person‐centred care. We used standard multiple regression analyses to assess the explained variance of patient safety culture dimensions on person‐centred care.

All regressions analyses were conducted separately for the nursing homes and home care services samples, as well as in total. The *p*‐value was set to .05. Preliminary analyses were conducted to ensure no violations of the assumptions for conducting the regression analyses. Due to issues with multicollinearity and suppression effects (Shieh, 2006), the two dimensions “handoffs” and “management support and organizational learning” were excluded from the regression analyses with the patient safety culture dimensions as independent variables. The scatter plots indicated a good linear relationship between the study variables, and the Q–Q plots indicated normal distribution of each variable in the two samples. The Durbin–Watson *d* was 2.00 in the first regression analysis and 1.93 in the second regression analysis, which is between the two critical values of 1.5 < *d* < 2.5 (Ho, 2013), indicating no linear autocorrelations in the samples. Multicollinearity was tested by means of tolerance and VIF values, which indicated no violations of this assumption. Normality of residuals was tested with a normal P–P plot, where the results indicated that the residuals were normally distributed. Occupational status and years of employment were controlled for in all analyses.

## 3 RESULTS

### 3.1 Person‐centred care, transformational leadership, job demands and job resources

In a hierarchical multiple regression analysis of person‐centred care in both nursing homes and home care, the full model with transformational leadership, job demands and job resources as predictors explained 41% of the variance in person‐centred care, with work pace as the strongest predictor (*β* = 0.39; *p* < .001; Table 3). The full model explained 45.7% in nursing homes and 40.4% in home care services. Transformational leadership was the strongest predictor in nursing homes (*β* = 0.39 *p* < .001), followed by work pace (*β* = 0.21 *p* < .05). In home care, work pace was the strongest predictor (*β* = 0.53 *p* < .001), followed by transformational leadership (*β* = 0.19 *p* < .05). Of the job resources, autonomy was a significant predictor in nursing homes, while participation was a significant predictor in home care services.

**TABLE 3:** Hierarchical multiple regression analyses of person‐centred care with transformational leadership, job demands and job resources as predictors

	Person‐centred care										
	Occupational status	Years employed	Leadership	Work pace	Mental workload	Emotional workload	Skill utilization	Autonomy	Participation	*R* ^2^	*F* for change in *R* ^2^
	*β*	*β*	*β*	*β*	*β*	*β*	*β*	*β*	*β*		
Nursing homes (*N* = 165)											
Model 1	−052	0.149								.010	1.78
Model 2	0.013	0.134*	0.579**							.340	82.19**
Model 3	−017	0.076	0.484**	−0.258*	0.068	−0.165*				.420	8.34**
Model 4	0.026	0.063	0.390**	−0.207*	0.000	−0.118	0.117	0.188*	0.005	.457	4.58*
Home care (*N* = 139)											
Model 1	0.048	−0.223*								.037	3.67*
Model 2	0.049	−0.124	0.374**							.163	21.49**
Model 3	−0.029	−0.119	0.292**	−0.603**	0.205*	0.002				.390	17.69**
Model 4	0.000	−0.120	0.191*	−0.534**	0.157	0.017	0.051	−0.021	0.188*	.403	2.02
Total (*N* = 304)											
Model 1	0.007	0.004								−.007	0.011
Model 2	0.025	0.054	0.512**							.251	104.61**
Model 3	−0.037	0.010	0.400**	−0.399**	0.111	−0.097				.379	21.66**
Model 4	0.006	0.008	0.292**	−0.339**	0.050	−0.048	0.093	0.093	0.113	.410	6.12**

* *p* < .05

** *p* < .001

### 3.2 Person‐centred care and patient safety culture

In a multiple regression analysis of person‐centred care in both nursing homes and home care, the full model with patient safety culture dimensions as predictors explained 57.5% of the variance in person‐centred care, with staffing as the strongest predictor (*β* = 0.31 *p* < .001; Table 4). The full model explained 64.5% in nursing homes and 51.6% in home care services. Feedback and communication about incidents was the strongest predictor in nursing homes (*β* = 0.27 *p* < .001), followed by staffing and supervisor expectations. In home care, staffing was the strongest predictor (*β* = 0.36 *p* < .001), followed by feedback and communication about incidents, and communication openness.

**TABLE 4:** Multiple regression analyses of person‐centred care with patient safety culture dimensions as predictors

Variables	Person‐centred care								
	Nursing homes (*N* = 165)			Home care (*N* = 139)			Total (*N* = 304)		
	*B*	SE	*β*	*B*	SE	*β*	*B*	SE	*β*
Teamwork	−0.004	0.058	−0.005	0.028	0.068	0.032	0.002	0.041	0.002
Staffing	0.243	0.058	0.254**	0.289	0.060	0.357**	0.272	0.041	0.306**
Compliance with procedures	−0.024	0.054	−0.027	0.067	0.063	0.075	0.012	0.039	0.013
Training and skills	0.111	0.059	0.139	−0.015	0.071	−0.017	0.061	0.044	0.075
Non‐punitive responses to mistakes	0.089	0.066	0.095	−0.016	0.074	−0.016	0.042	0.047	0.043
Feedback and communication about incidents	0.256	0.067	0.273**	0.201	0.075	0.212*	0.219	0.049	0.233**
Communication openness	0.077	0.056	0.103	0.182	0.071	0.232*	0.122	0.042	0.160*
Supervisor expectations	0.136	0.047	0.189*	0.071	0.069	0.092	0.132	0.037	0.179**
*R* ^2^			.645**			.516**			.575**

* *p* < .05

** *p* < .001.

## 4 DISCUSSION

This study indicates that transformational leadership, work pace and patient safety culture dimensions related to staffing and communication are important predictors for person‐centred care in nursing homes and home care services. As hypothesized, transformational leadership and job resources positively predicted person‐centred care, while job demands had a negative impact. However, although the job resource autonomy was a significant predictor in nursing homes, and participation predicted person‐centred care in home care services, none of the job resources had a significant impact in total. Furthermore, the job demand “work pace” was a stronger predictor for person‐centred care than transformational leadership. The findings are in line with a recent mixed methods study in Norwegian nursing homes and home care, showing that lack of communication and information exchange between healthcare services, users and next of kin are among the main challenges for person‐centred care (Ree, Wiig, Braithwaite, & Aase, 2020). The study also emphasizes challenges related to busy schedules and poor staffing (Ree et al., 2020). Poor staffing is repeatedly found being related to a range of negative factors in health care, such as workload, employee burnout, work dissatisfaction, and poor patient safety and quality of care (Cho et al., 2016; Shin, Park, & Bae, 2018), and being a barrier for person‐centred care (Engle et al., 2017). Higher staffing levels and resource adequacy on the other hand are related to higher levels of person‐centred care (Bachnick, Ausserhofer, Baernholdt, & Simon, 2018). Similar to our findings, Nkrumah and Abekah‐Nkrumah (2019) found that leadership commitment and support were important facilitators for person‐centred care, while communication challenges acted as barriers.

There is probably an interaction and mutual influence between the predictive factors work pace, staffing and communication, as poor staffing will lead to a more busy work schedule with higher work pace and higher likelihood of communication gaps (Yanchus, Ohler, Crowe, Teclaw, & Osatuke, 2017). Several studies report that work environmental factors such as feeling overworked, staff shortages, lack of time and tools, and workload are obstacles for patient‐centred care (van Mol et al., 2017; Ocloo et al., 2020; West, Barron, & Reeves, 2005). This might partly be because employees’ stress levels and workload reduce their possibilities of spending time with and connect with the patients (Bishop & Macdonald, 2014). Previous studies show that managers influence the patient safety culture through their impact on work environmental factors (Boamah et al., 2018; Weng, Kim, & Wu, 2017), and based on our findings we can assume the same accounts for person‐centred care. The strong influence of transformational leadership on person‐centred care in our study indicates that managers play an important role in facilitating for a culture where person‐centred care is possible to implement and practice. A number of studies show that the staff–patient relationship, having enough time to get to know the patients and good communication among employees, staff and next of kin are all key for patient‐centred care (Angel & Frederiksen, 2015; Oxelmark, Ulin, Chaboyer, Bucknall, & Ringdal, 2018; Ree et al., 2020; Vennik, van de Bovenkamp, Putters, & Grit, 2016). Thus, for person‐centred care practices to be sustainable over time, managers need to create a work environment with a proper balance between job demands and resources, ensuring apt staffing levels and communication channels that enable employees to make person‐centred care a part of daily practice. Previous research in nursing homes and home care has shown that although external contextual factors such as policy and guidelines, and financial resources influence these work environmental factors, managers are key in acting upon, adapting and negotiating the context to facilitate sound healthcare quality (Johannessen, Ree, Aase, Bal, & Wiig, 2020; Ree, Johannessen, & Wiig, 2019).

### 4.1 Strengths and limitations

The main strength of this study is its originality in being the first to explore the role of patient safety culture, transformational leadership and work environmental factors for person‐centred care in nursing home and home care settings. The diversity in the samples regarding location and size of units and municipalities is representative for this sector in Norway and provides different nuances and perspectives in the data material. The majority of females in the samples is also representative for these settings in general across the country.

The main limitation is the cross‐sectional design. Since all variables are measured at the same point in time, caution must be made when drawing inferences about cause and effects. Furthermore, the samples were too small to conduct valid sub‐group analyses. Future studies should examine the role of transformational leadership, work environmental factors and patient safety culture on person‐centred care in longitudinal studies, and explore differences between different sub‐groups in the target population, such as age groups, occupational status and across departments.

## 5 CONCLUSIONS AND IMPLICATIONS

Transformational leadership, job demands, staffing and communication seem to be important factors for person‐centred care in nursing homes and home care services. High pace of work (having to work very fast, time pressure) reduce the utilization of person‐centred care, while transformational leadership, sound staffing and good communication increase it. These findings have several implications for nursing management. Lack of resources such as staffing, time and money for training and necessary support tools, might hinder managers’ room for manoeuver and possibilities to implement the actions required to promote person‐centred care. Policy guidelines and regulations should emphasize the importance of transformational leadership, work environmental factors and patient safety culture for person‐centred care. Policymakers need to be aware of the importance of allocating sufficient resources for nursing homes and home care services to facilitate person‐centred care practices. From an organizational perspective, interventions should focus on training and education of managers in the use of transformational leadership behaviours, training and competence development among managers and staff in person‐centred care approaches, and how to utilize information gained from users to improve practice. It is important to ensure sufficient staff levels and professional competence in the workforce to safeguard person‐centred care practices through proper systems and routines and to establish good communication channels across all system levels. Organizations should make time and room for reflexive spaces (Wiig, Aase, & Bal, 2020) where management teams can discuss and reflect upon workplace issues and practices, including how to align available resources with demands, and how to prioritize and work to enable person‐centred care. Employees should be regularly involved in such discussions and reflections, ensuring good communication within and across departments and units. Communication and collaboration with networks, policymakers and resource persons outside the units can promote learning contributing to improvement of person‐centred care practices (Johannessen et al., 2020; Ree et al., 2019, 2020). Furthermore, managers should have access to and make use of support tools, for example surveys to map user experiences and opinions that can be used to inform and improve practice, ensuring that the services are aligned with the needs and preferences of the users and patients in the nursing homes and home care services.

## CONFLICT OF INTEREST

None declared.

## ETHICS APPROVAL AND CONSENT TO PARTICIPATE

The Regional Committees for Research Ethics in Norway regarded the study to not be governed by the Health Research Act and was therefore not within their mandate. The study was approved by the Norwegian Social Science Data Services (NSD, ID 52324) and followed the principles from the Helsinki Declaration. All participants gave their written informed consent at the very beginning of the electronic questionnaire, where it was stated that they consented to participate by responding to the questionnaire. Participants were informed that their responses to the questionnaire were confidential and only available to the researchers.

## Data Availability

The data sets used during the current study are available from the corresponding author on reasonable request.

## References

[nop2592-bib-0001] Abrahamson, K. , Hass, Z. , Morgan, K. , Fulton, B. , & Ramanujam, R. (2016). The relationship between nurse‐reported safety culture and the patient experience. Journal of Nursing Administration, 46(12), 662–668. 10.1097/NNA.0000000000000423 27851708

[nop2592-bib-0002] Angel, S. , & Frederiksen, K. N. (2015). Challenges in achieving patient participation: A review of how patient participation is addressed in empirical studies. International Journal of Nursing Studies, 52(9), 1525–1538. 10.1016/j.ijnurstu.2015.04.008 25980558

[nop2592-bib-0003] Bachnick, S. , Ausserhofer, D. , Baernholdt, M. , & Simon, M. (2018). Patient‐centered care, nurse work environment and implicit rationing of nursing care in Swiss acute care hospitals: A cross‐sectional multi‐center study. International Journal of Nursing Studies, 81, 98–106. 10.1016/j.ijnurstu.2017.11.007 29554590

[nop2592-bib-0004] Bass, B. M. (1991). From transactional to transformational leadership: Learning to share the vision. Organizational Dynamics, 18(3), 19–31. 10.1016/0090-2616(90)90061-S

[nop2592-bib-0005] Beckett, P. , Field, J. , Molloy, L. , Yu, N. , Holmes, D. , & Pile, E. (2013). Practice what you preach: Developing person‐centred culture in inpatient mental health settings through strengths‐based, transformational leadership. Issues in Mental Health Nursing, 34(8), 595–601. 10.3109/01612840.2013.790524 23909671

[nop2592-bib-0006] Bishop, A. C. , & Macdonald, M. (2014). Patient involvement in patient safety: A qualitative study of nursing staff and patient perceptions. Journal of Patient Safety, 13(2), 82–87. 10.1097/PTS.0000000000000123 25010194

[nop2592-bib-0007] Boamah, S. A. , Laschinger, H. K. S. , Wong, C. , & Clarke, S. (2018). Effect of transformational leadership on job satisfaction and patient safety outcomes. Journal of Nursing Outlook, 66(2), 180–189. 10.1016/j.outlook.2017.10.004 29174629

[nop2592-bib-0008] Braithwaite, J. , Herkes, J. , Ludlow, K. , Testa, L. , & Lamprell, G. (2017). Association between organisational and workplace cultures, and patient outcomes: Systematic review. British Medical Journal Open, 7(11), e017708 10.1136/bmjopen-2017-017708 PMC5695304 29122796

[nop2592-bib-0009] Cappelen, K. , Aase, K. , Storm, M. , Hetland, J. , & Harris, A. (2016). Psychometric properties of the Nursing Home Survey on Patient Safety Culture in Norwegian nursing homes. BMC Health Services Research, 16(1), 446 10.1186/s12913-016-1706-x 27567673 PMC5002111

[nop2592-bib-0010] Carless, S. A. , Wearing, A. J. , & Mann, L. (2000). A short measure of transformational leadership. Journal of Business Psychology, 14(3), 389–405.

[nop2592-bib-0011] Cho, E. , Lee, N. , Kim, E. , Kim, S. , Lee, K. , Park, K. , & Sung, Y. H. (2016). Nurse staffing level and overtime associated with patient safety, quality of care, and care left undone in hospitals: A cross‐sectional study. International Journal of Nursing Studies, 60, 263–271. 10.1016/j.ijnurstu.2016.05.009 27297386

[nop2592-bib-0012] Coulter, A. (2006). Engaging patients in healthcare. How is the UK doing relative to other countries? Oxford: Picker Institute Europe.

[nop2592-bib-0013] Demerouti, E. , Bakker, A. B. , Nachreiner, F. , & Schaufeli, W. (2001). The job demands‐resources model of burnout. Journal of Applied Psychology, 86(3), 499 10.1037/0021-9010.86.3.499 11419809

[nop2592-bib-0014] Doyle, C. , Lennox, L. , & Bell, D. (2013). A systematic review of evidence on the links between patient experience and clinical safety and effectiveness. British Medical Journal Open, 3(1), e001570 10.1136/bmjopen-2012-001570 PMC3549241 23293244

[nop2592-bib-0015] Edvardsson, D. , Fetherstonhaugh, D. , Nay, R. , & Gibson, S. (2010). Development and initial testing of the Person‐centered Care Assessment Tool (P‐CAT). International Psychogeriatrics, 22(1), 101–108. 10.1017/S1041610209990688 19631005

[nop2592-bib-0016] Engle, R. L. , Tyler, D. A. , Gormley, K. E. , Afable, M. K. , Curyto, K. , Adjognon, O. L. , … Sullivan, J. L. (2017). Identifying barriers to culture change: A qualitative analysis of the obstacles to delivering resident‐centered care. Psychological Services, 14(3), 316 10.1037/ser0000169 28805416

[nop2592-bib-0017] Goldfarb, M. J. , Bibas, L. , Bartlett, V. , Jones, H. , & Khan, N. (2017). Outcomes of patient‐and family‐centered care interventions in the ICU: A systematic review and meta‐analysis. Critical Care Medicine, 45(10), 1751–1761. 10.1097/CCM.0000000000002624 28749855

[nop2592-bib-0018] Ho, R. (2013). Handbook of univariate and multivariate data analysis with IBM SPSS. Boca Raton: CRC Press.

[nop2592-bib-0019] Institute of Medicine (2001). Crossing the Quality Chasm: A New Health System for the 21st Century. Washington, D.C.: National Academies Press (US). 25057539

[nop2592-bib-0020] Johannessen, T. , Ree, E. , Aase, I. , Bal, R. , & Wiig, S. (2020). Exploring challenges in quality and safety work in nursing homes and home care – A case study as basis for theory development. BMC Health Services Research, 20(1), 45 10.1186/s12913-020-05149-x 32245450 PMC7118914

[nop2592-bib-0021] Johannessen, T. , Ree, E. , Strømme, T. , Aase, I. , Bal, R. , & Wiig, S. (2019). Designing and pilot testing of a leadership intervention to improve quality and safety in nursing homes and home care (the SAFE‐LEAD intervention). British Medical Journal Open, 9(6), 10.1136/bmjopen-2018-027790 PMC6597165 31213451

[nop2592-bib-0022] Kim, S. K. , & Park, M. (2017). Effectiveness of person‐centered care on people with dementia: A systematic review and meta‐analysis. Clinical Interventions in Aging, 12, 381–397. 10.2147/CIA.S117637 28255234 PMC5322939

[nop2592-bib-0023] Kiwanuka, F. , Shayan, S. J. , & Tolulope, A. A. (2019). Barriers to patient and family‐centred care in adult intensive care units: A systematic review. Nursing Open, 6(3), 676–684. 10.1002/nop2.253 31367389 PMC6650666

[nop2592-bib-0024] Merrill, K. C. (2015). Leadership style and patient safety: Implications for nurse managers. Journal of Nursing Administration, 45(6), 319–324. 10.1097/NNA.0000000000000207 26010281

[nop2592-bib-0025] Ministry of Health and Care Services . (2018). Meld. St. 15 (2017–2018). Leve hele livet ‐ En kvalitetsreform for eldre [in Norwegian]. Oslo: Ministry of Health and Care Services.

[nop2592-bib-0026] Moore, L. , Britten, N. , Lydahl, D. , Naldemirci, Ö. , Elam, M. , & Wolf, A. (2017). Barriers and facilitators to the implementation of person‐centred care in different healthcare contexts. Scandinavian Journal of Caring Sciences, 31(4), 662–673. 10.1111/scs.12376 27859459 PMC5724704

[nop2592-bib-0027] Najjar, S. , Nafouri, N. , Vanhaecht, K. , & Euwema, M. (2015). The relationship between patient safety culture and adverse events: A study in Palestinian hospitals. Safety in Health, 1(1), 16 10.1186/s40886-015-0008-z

[nop2592-bib-0028] Nielsen, M. B. , Skogstad, A. , Gjerstad, J. , & Einarsen, S. V. (2019). Are transformational and laissez‐faire leadership related to state anxiety among subordinates? A two‐wave prospective study of forward and reverse associations. Work & Stress, 33(2), 137–155. 10.1080/02678373.2018.1528307

[nop2592-bib-0029] Nkrumah, J. , & Abekah‐Nkrumah, G. (2019). Facilitators and barriers of patient‐centered care at the organizational‐level: A study of three district hospitals in the central region of Ghana. BMC Health Services Research, 19(1), 1–11. 10.1186/s12913-019-4748-z 31775809 PMC6882059

[nop2592-bib-0030] Notelaers, G. , De Witte, H. , Van Veldhoven, M. , & Vermunt, J. K. (2007). Construction and validation of the short inventory to monitor psychosocial hazards. Médecine Du Travail Et Ergonomie, 44, 11–17.

[nop2592-bib-0031] Ocloo, J. , Goodrich, J. , Tanaka, H. , Birchall‐Searle, J. , Dawson, D. , & Farr, M. (2020). The importance of power, context and agency in improving patient experience through a patient and family centred care approach. Health Research Policy and Systems, 18(1), 10 10.1186/s12961-019-0487-1 31973712 PMC6979369

[nop2592-bib-0032] Oxelmark, L. , Ulin, K. , Chaboyer, W. , Bucknall, T. , & Ringdal, M. (2018). Registered Nurses’ experiences of patient participation in hospital care: Supporting and hindering factors patient participation in care. Scandinavian Journal of Caring Sciences, 32(2), 612–621. 10.1111/scs.12486 28675925

[nop2592-bib-0033] Rathert, C. , Wyrwich, M. D. , & Boren, S. A. (2013). Patient‐centered care and outcomes: A systematic review of the literature. Medical Care Research and Review, 70(4), 351–379. 10.1177/1077558712465774 23169897

[nop2592-bib-0034] Ree, E. , Johannessen, T. , & Wiig, S. (2019). How do contextual factors influence quality and safety work in the Norwegian home care and nursing home settings? A qualitative study about managers’ experiences. British Medical Journal Open, 9(7), 10.1136/bmjopen-2018-025197 PMC6629381 31289055

[nop2592-bib-0035] Ree, E. , & Wiig, S. (2019a). Employees’ perceptions of patient safety culture in Norwegian nursing homes and home care services. BMC Health Services Research, 19(1), 1–7. 10.1186/s12913-019-4456-8 31464630 PMC6716833

[nop2592-bib-0036] Ree, E. , & Wiig, S. (2019b). Linking transformational leadership, patient safety culture and work engagement in home care services. Nursing Open, 7(1), 256–264. 10.1002/nop2.386 31871709 PMC6917935

[nop2592-bib-0037] Ree, E. , Wiig, S. , Braithwaite, J. , & Aase, I. (2020). To what degree and how do healthcare professionals in nursing homes and homecare practice user involvement? A mixed methods study. Tidsskrift for Omsorgsforskning (in review).

[nop2592-bib-0038] Rokstad, A. M. M. , Engedal, K. , Edvardsson, D. , & Selbæk, G. (2012). Psychometric evaluation of the Norwegian version of the Person‐centred Care Assessment Tool. International Journal of Nursing Practice, 18(1), 99–105. 10.1111/j.1440-172X.2011.01998.x 22257337

[nop2592-bib-0039] Sfantou, D. , Laliotis, A. , Patelarou, A. , Sifaki‐Pistolla, D. , Matalliotakis, M. , & Patelarou, E. (2017). Importance of leadership style towards quality of care measures in healthcare settings: A systematic review. Healthcare, 5(4), 73 10.3390/healthcare5040073 PMC5746707 29036901

[nop2592-bib-0040] Shieh, G. (2006). Suppression situations in multiple linear regression. Educational and Psychological Measurement, 66(3), 435–447. 10.1177/0013164405278584

[nop2592-bib-0041] Shin, S. , Park, J.‐H. , & Bae, S.‐H. (2018). Nurse staffing and nurse outcomes: A systematic review and meta‐analysis. Nursing Outlook, 66(3), 273–282. 10.1016/j.outlook.2017.12.002 29685321

[nop2592-bib-0042] Sorra, J. , Franklin, M. , & Streagle, S. (2008). Nursing home survey on patient safety culture. Rockville, MD: Agency for Healthcare Research and Quality.

[nop2592-bib-0043] van Mol, M. M. , Boeter, T. G. , Verharen, L. , Kompanje, E. J. , Bakker, J. , & Nijkamp, M. D. (2017). Patient‐and family‐centred care in the intensive care unit: A challenge in the daily practice of healthcare professionals. Journal of Clinical Nursing, 26(19–20), 3212–3223. 27875001 10.1111/jocn.13669

[nop2592-bib-0044] Vennik, F. D. , van de Bovenkamp, H. M. , Putters, K. , & Grit, K. J. (2016). Co‐production in healthcare: Rhetoric and practice. International Review of Administrative Sciences, 82(1), 150–168. 10.1177/0020852315570553

[nop2592-bib-0045] Wang, X. , Liu, K. , You, L. , Xiang, J. , Hu, H. , Zhang, L. , … Zhu, X. (2014). The relationship between patient safety culture and adverse events: A questionnaire survey. International Journal of Nursing Studies, 51(8), 1114–1122. 10.1016/j.ijnurstu.2013.12.007 24418106

[nop2592-bib-0046] Weng, S. , Kim, S. , & Wu, C. (2017). Underlying influence of perception of management leadership on patient safety climate in healthcare organizations–A mediation analysis approach. International Journal for Quality in Health Care, 29(1), 111–116. 27920245 10.1093/intqhc/mzw145

[nop2592-bib-0047] West, E. , Barron, D. N. , & Reeves, R. (2005). Overcoming the barriers to patient‐centred care: Time, tools and training. Journal of Clinical Nursing, 14(4), 435–443. 10.1111/j.1365-2702.2004.01091.x 15807750

[nop2592-bib-0048] Wiig, S. , Aase, K. , & Bal, R. (2020). Reflexive spaces: Leveraging resilience into healthcare regulation and management. Journal of Patient Safety. 10.1097/PTS.0000000000000658 PMC8612922 32011428

[nop2592-bib-0049] Wiig, S. , Ree, E. , Johannessen, T. , Strømme, T. , Storm, M. , Aase, I. , … Aase, K. (2018). Improving quality and safety in nursing homes and home care: The study protocol of a mixed‐methods research design to implement a leadership intervention. British Medical Journal Open, 8(3), e020933 10.1136/bmjopen-2017-020933 PMC5875614 29599394

[nop2592-bib-0050] Wijk, K. , Åberg Jönsson, F. , & Lindberg, M. (2019). Perceived enabling factors and barriers for the implementation of improvements in health care in order to achieve patient‐centred care: A case report from Sweden. Journal of Evaluation in Clinical Practice, 26(3), 791–800. 10.1111/jep.13272 31475435

[nop2592-bib-0051] World Health Organization (2016). Framework on integrated, people‐centred health services. Report by the Secretariat. Geneva: World Health Organization Retrieved from https://apps.who.int/gb/ebwha/pdf_files/WHA69/A69_39‐en.pdf?ua=1

[nop2592-bib-0052] Yanchus, N. J. , Ohler, L. , Crowe, E. , Teclaw, R. , & Osatuke, K. (2017). ‘You just can’t do it all’: A secondary analysis of nurses' perceptions of teamwork, staffing and workload. Journal of Research in Nursing, 22(4), 313–325. 10.1177/1744987117710305

